# Data Sources for Drug Utilization Research in Brazil—DUR-BRA Study

**DOI:** 10.3389/fphar.2021.789872

**Published:** 2022-01-14

**Authors:** Lisiane Freitas Leal, Claudia Garcia Serpa Osorio-de-Castro, Luiz Júpiter Carneiro de Souza, Felipe Ferre, Daniel Marques Mota, Marcia Ito, Monique Elseviers, Elisangela da Costa Lima, Ivan Ricardo Zimmernan, Izabela Fulone, Monica Da Luz Carvalho-Soares, Luciane Cruz Lopes

**Affiliations:** ^1^ Department of Epidemiology, Biostatistics and Occupational Health, McGill University, Montreal, QC, Canada; ^2^ Sergio Arouca National School of Public Health, Oswaldo Cruz Foundation, Rio de Janeiro, Brazil; ^3^ Regional Management, Oswaldo Cruz Foundation, Brasília, Brazil; ^4^ Faculdade de Medicina, Programa de Pós-Graduação em Saúde Pública, Universidade Federal de Minas Gerais, Belo Horizonte, Brazil; ^5^ Brazilian Health Regulatory Agency, Brasília, Brazil; ^6^ Professional Master’s Program in Productive Systems, Centro Estadual de Educação Tecnológica Paula Souza, São Paulo, Brazil; ^7^ Ghent University, Ghent, Belgium; ^8^ Faculdade de Farmácia, Universidade Federal do Rio de Janeiro, Rio de Janeiro, Brazil; ^9^ Faculdade de Ciências da Saúde Campus Darcy Ribeiro, University of Brasília, Brasília, Brazil; ^10^ Graduate Pharmaceutical Science, University of Sorocaba, Sao Paulo, Brazil

**Keywords:** pharmacoepidemiology, health information systems, databases (all types), Brazil, database management systems, pharmaceutical preparations, data sources, drug utilisation research

## Abstract

**Background:** In Brazil, studies that map electronic healthcare databases in order to assess their suitability for use in pharmacoepidemiologic research are lacking. We aimed to identify, catalogue, and characterize Brazilian data sources for Drug Utilization Research (DUR).

**Methods:** The present study is part of the project entitled, “Publicly Available Data Sources for Drug Utilization Research in Latin American (LatAm) Countries.” A network of Brazilian health experts was assembled to map secondary administrative data from healthcare organizations that might provide information related to medication use. A multi-phase approach including internet search of institutional government websites, traditional bibliographic databases, and experts’ input was used for mapping the data sources. The reviewers searched, screened and selected the data sources independently; disagreements were resolved by consensus. Data sources were grouped into the following categories: 1) automated databases; 2) Electronic Medical Records (EMR); 3) national surveys or datasets; 4) adverse event reporting systems; and 5) others. Each data source was characterized by accessibility, geographic granularity, setting, type of data (aggregate or individual-level), and years of coverage. We also searched for publications related to each data source.

**Results:** A total of 62 data sources were identified and screened; 38 met the eligibility criteria for inclusion and were fully characterized. We grouped 23 (60%) as automated databases, four (11%) as adverse event reporting systems, four (11%) as EMRs, three (8%) as national surveys or datasets, and four (11%) as other types. Eighteen (47%) were classified as publicly and conveniently accessible online; providing information at national level. Most of them offered more than 5 years of comprehensive data coverage, and presented data at both the individual and aggregated levels. No information about population coverage was found. Drug coding is not uniform; each data source has its own coding system, depending on the purpose of the data. At least one scientific publication was found for each publicly available data source.

**Conclusions:** There are several types of data sources for DUR in Brazil, but a uniform system for drug classification and data quality evaluation does not exist. The extent of population covered by year is unknown. Our comprehensive and structured inventory reveals a need for full characterization of these data sources.

## Introduction

Drug utilization research (DUR) aims to examine patterns of medication use and adherence to treatments and to assess determinants of utilization (Godman et al., 2016; Wettermark et al., 2016) The history of DUR is described elsewhere (World Health Organization, 1993; World Health Organization, 2003b; Wettermark, 2013; Wettermark et al., 2016). Over the years, the scope of DUR has expanded; methods have improved, and the use of secondary data has increased. Nonetheless, additional work is required, particularly with regard to the quality of available data (Evans, 2012; Schneeweiss, 2019).

Secondary data that are used for pharmacoepidemiology research are usually derived from information routinely collected for administrative purposes and as part of patient care (Eriksson and Ibáñez, 2016), such as drug sales, medical billing, and prescriptions (Shalini et al., 2010). Given the cost and difficulty of primary data collection, electronic healthcare databases (EHD) are commonly used in many countries to study drug safety (Pacurariu et al., 2018). Linkage of data on medication use with diagnostic, mortality, and other health databases has become routine in Europe, North America, and Asian countries (Wettermark, 2013), but not in low- and middle-income countries, notably, in Latin America (de Castro, 1999; de Castro, 2000; World Health Organization, 2003a; Baldoni, 2011; Coelho and Santos, 2012).

While high-income countries are leveraging the use of Real-World Evidence to inform regulatory decision-making (European Medicines Agency (EMA), 2018; Health Canada, 2019; Food and Drug Administration (FDA), 2020), in Latin America initiatives are incipient and limited to few settings (Durán et al., 2016; Salas et al., 2018). In Brazil, efforts related to “open data” have improved the prospects for creating systematic approaches to the use of secondary data, not only for decision-making but also for research (Controladoria Geral da União, 2020).

Despite awareness of the value of existing databases, and observed expansion of DUR in Brazil using secondary data, a mapping of databases to evaluate their potential, as well as their characteristics and applications, has not been undertaken.

The present work aimed, therefore, to identify, catalogue, and characterize secondary data sources for DUR in Brazil.

## Methods

### Design

This project was derived from the “Publicly Available Data Sources for Drug Utilization Research in Latin American (LatAm) Countries—DASDURLATAM study,” which is an initiative supported by the International Society for Pharmacoepidemiology (ISPE) to make an inventory for all LatAm countries (Lopes et al., 2021).

We employed a multi-phase approach to map Brazilian data sources. A network of national health experts was assembled to prepare an initial inventory of data sources for DUR. A multidisciplinary network was established. Fourteen Brazilian researchers experts in pharmacoepidemiology and health professionals working in both academia and the government sector were invited and accepted to participate. A pharmacoepidemiology expert in European data sources for DUR joined the Brazilian team (ME). A literature review was conducted to retrieve drug utilization studies conducted in Brazil using secondary data. Finally, data sources were selected and characterized.

### Type of Data Sources (Eligibility Criteria)

The eligibility criteria for inclusion in the inventory specified Brazilian data sources generated by healthcare organizations that provide information related to medication use. Data sources from health insurance companies or other commercial providers (e.g., IQVIA) were not eligible. The Brazilian health care system consists of public and private components. Population access depends on several factors, including the ability to pay for health care. We, therefore, focused on data sources generated by the public health system because:

1) The public system provides national data with municipality granularity.
2) Almost 80% of the Brazilian population is covered by the public system; private health care insurance companies are spread across the country and comprise many small companies, not representative of the general population (Paim et al., 2011; Massuda et al., 2018).
3) It is not possible to map data with no payment requests or ethical approval.

We excluded data sources in which information about medicines (names or codes) was not recorded.

### Search Strategy

We conducted an internet search of institutional government websites up to January 2021. To retrieve studies, we reviewed the literature available on traditional bibliographic databases (MEDLINE/PubMed, LILACS, Google Scholar) from inception to August 2020, with no limits on publication type, status, or language. The concept terms were freely combined, using Boolean operators (AND/OR): “pharmacoepidemiology,” “drug utilization,” “BRAZIL,” and the acronym of the data source first identified. The Systems and Products Catalog of the Informatics Department of the Unified Health System–DataSUS (Ministério da Saúde, 2020) was also reviewed. This was done to assess the description of all systems already available through the Ministry of Health interface, and the availability of medication data recorded by the Ministry of Health, and not previously identified by the network of specialists or through the literature review.

### Screening of Data Sources for Drug Utilization Research

Working in pairs and independently, the expert network (DMM, CGSOC, LCL, FF, LFL and LJCS) conducted in-depth screening and reviewed potentially eligible data sources. Disagreements on whether specific data sources contained drug information and whether they should remain on the list to be mapped as potential data source for DUR were discussed in online meetings. A consensus was achieved on the inclusion or exclusion of data sources.

### Data Collection and Data Analysis

The data sources were classified and grouped into the following categories: 1) automated databases (subclassified as administrative claims data and other transactional and operational data); 2) Electronic Medical Records (EMR); 3) national surveys or datasets; 4) adverse event reporting systems; and 5) other sources, according to Harpe et al.‘s classification for secondary data (Harpe, 2010) (Supplemental Table S1). For a general description of each data source, we used a seven-criteria checklist (Box 1). Additional information for characterizing the data sources was collected: custodian; data retrieval pathway, corresponding to the Uniform Resource Locator (URL) where the data source may be found; file format in which data are provided, that is, the way in which information is encoded for storage (comma-separated values—CSV, XLSX, ZIP, Plain Text-txt, or another format); and type of tables used for medication coding—European Article Number-EAN, Brazilian Non-proprietary Names (in Portuguese, *Denominação Comum Brasileira*—DCB), or other). Additional information was completed according to the provider’s definitions and specialist consultation (FF and LJCS). Each national DUR expert was responsible for reviewing the descriptions of the data sources and their final characterization.

## Results

**FIGURE 1:**
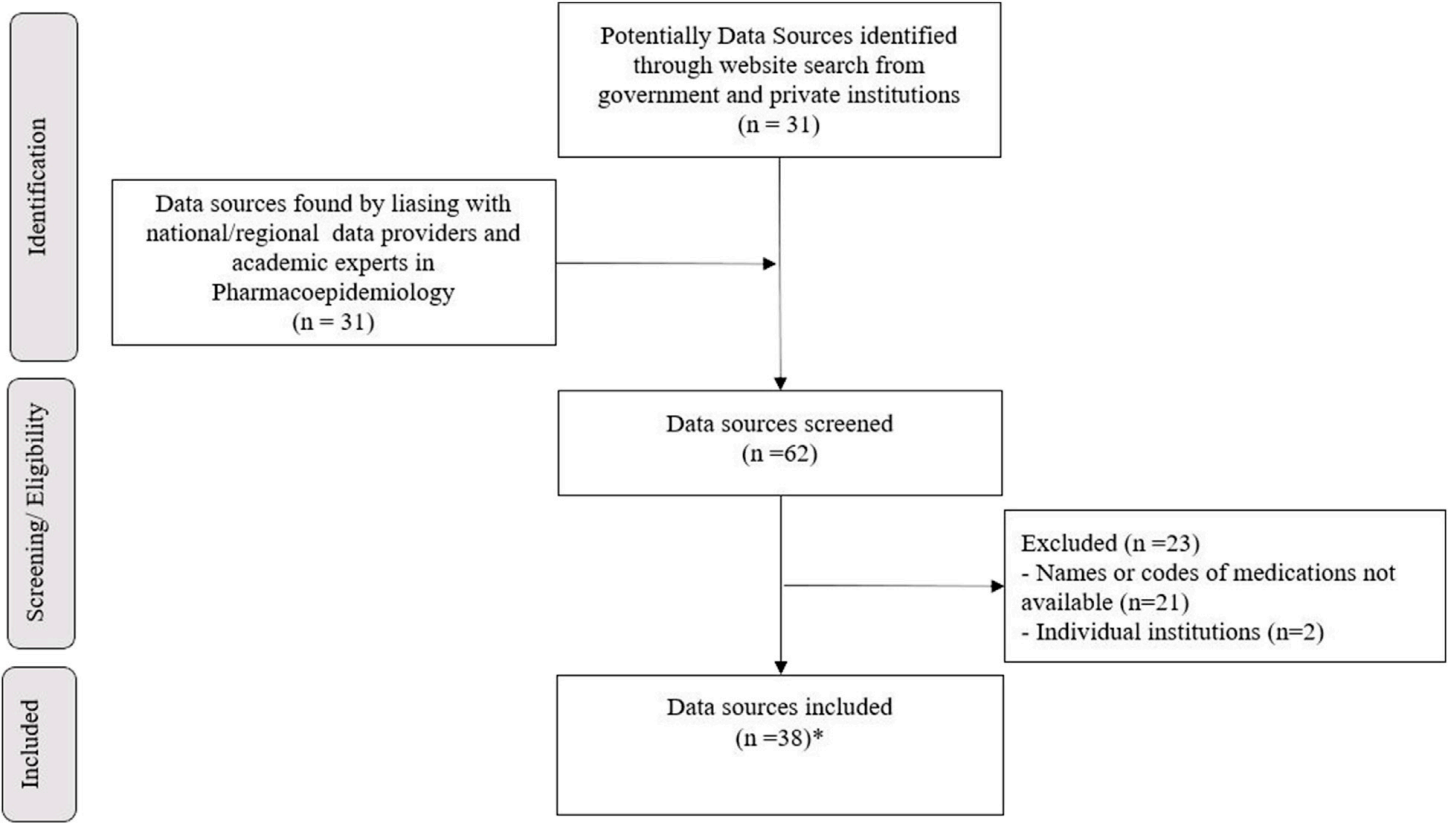
Data sources for DUR in Brazil selection flowchart. * means third-nine data sources were selected. When characterized, two were related to the same drug-purchasing system and were grouped into one data source (SIASG and SISME).

The expert network identified 62 data sources. After application of the exclusion criteria, 39 sources were included. Two of them (SIASG-*Sistema Integrado de Administração de Serviços Gerais* and SISME-*Sistema de Minuta de Empenho*) were related to the same drug-purchasing system and were grouped as one data source. Thus, the final selection consisted of 38 data sources, which underwent further characterization (Figure 1). Six rounds of discussion took place among the national health experts in order to achieve consensus and define the final list (Supplementary Table S2).

**FIGURE 2:**
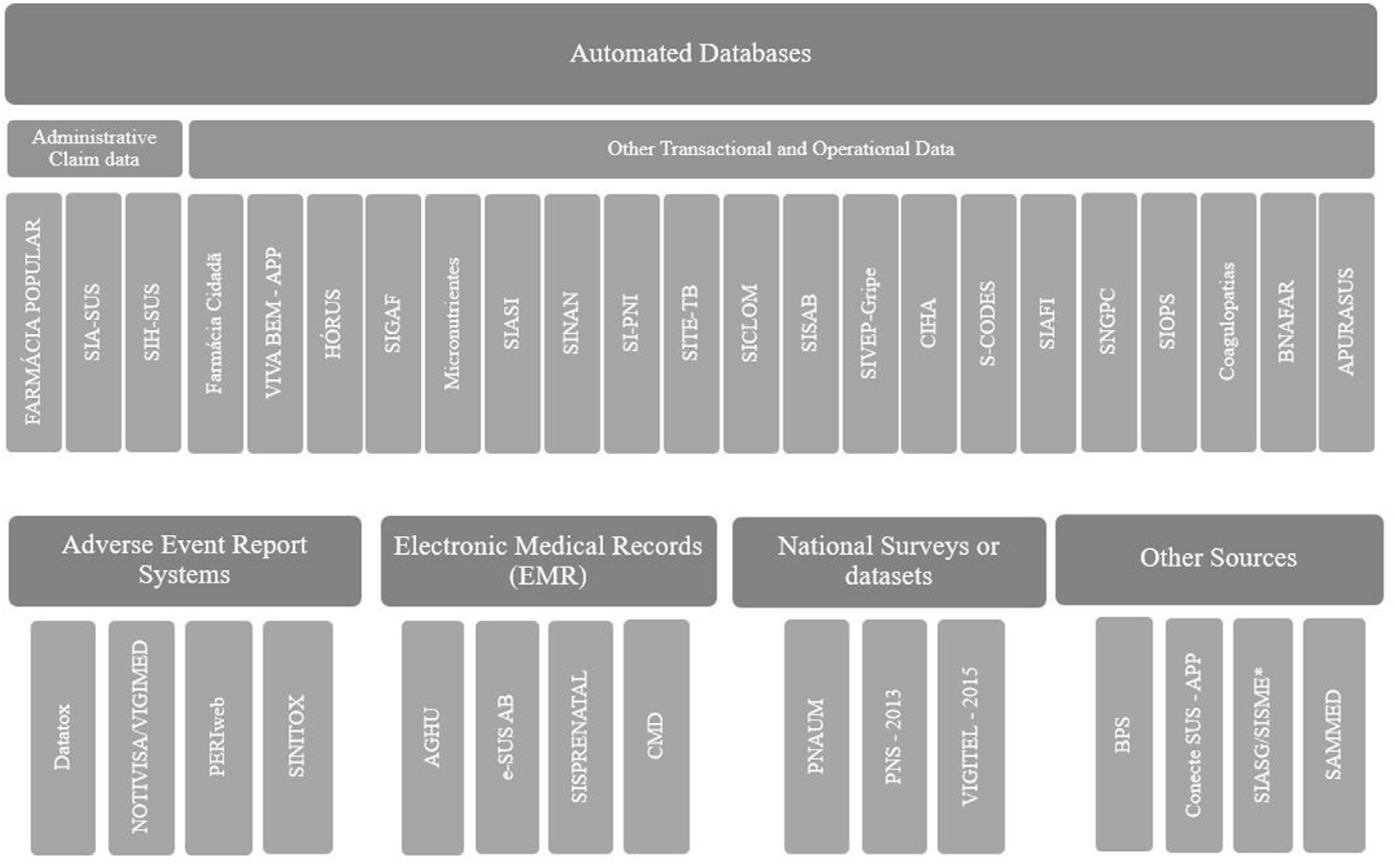
Classification of data sources identified in Brazil.

Figure 2 shows how the data sources were grouped. Twenty-three (60%) were classified as automated health care databases; four (11%) as EMRs; four (11%) as adverse event reporting systems; three (8%) as national surveys or datasets; and four (11%) as other types. The description of each data source, as well as the rationale for grouping it in a particular category, is provided in the supplementary material (Supplementary Table S3).

Based on the analysis of each data source, 18 (47%) were classified as “publicly and conveniently accessible online,” 15 of which (88%) were accessible through the DataSUS, with the Brazilian Ministry of Health as custodian. All publicly available online data sources provided national information; most of them had more than 5 years of coverage and both individual- and aggregate-level information. Twenty data sources (53%) were known to collect individual-level data, and three (PNAUM, SIA-SUS, and SIVEP-Gripe) were available for download. Table 1 displays the data sources, grouped by accessibility, geographic granularity, type, setting, and initial year of release. The detailed classification, which allows comparability among the data sources is provided in the supplementary material (Supplementary Table S4).

**TABLE 1:** Selected data sources, grouped by accessibility, geographic granularity, sector, setting, and type of data.

Characteristics	Data sources (N = 38)^a^
*Acessibility*	
*Publicly and conveniently accessible online*	BPS; CIHA; CMD; Micronutrientes; PNAUM; PNS; SI-PNI; SIA-SUS; SIH-SUS; SINAN; SINITOX; SIOPS; SISAB; SISPRENATAL; Sivep-gripe; SNGPC; and Vigitel
*Restricted pre-authorized protocol-only access*	AGHU
*Access limited to or dependent on country-specific legislation - Freedom of Information Act*	CIHA; e-SUS AB; Farmácia Cidadã; FARMÁCIA POPULAR; HORUS; NOTIVISA/VIGIMED; PERIweb; SIAFI; SIASG/SISME; SIA-SUS; SICLOM; SIGAF; SIH-SUS; SINAN; SISAB; SISPRENATAL; Site-TB; Sivep-gripe; and SNGPC
*Available only to researchers working in the institution (only people from the institution that provides the database)*	DATATOX; PERIweb; S-CODES; SIASG/SISME; and SIGAF
*The process for obtaining data is not clear, without general regulation*	None
*Not accessible/Data not available for public use*	APURASUS; BNAFAR; COAGULOPATIAS; Conecte SUS - APP; SAMMED; SIASI; and VIVA BEM - APP
***Geographic granularity***	
*National*	APURASUS; BNAFAR; BPS; CIHA; CMD; COAGULOPATIAS; DATATOX; e-SUS AB; FARMÁCIA POPULAR; HORUS; Micronutrientes; NOTIVISA/VIGIMED; PNAUM; PNS; SI-PNI; SIAFI; SIASG/SISME; SIA-SUS; SICLOM; SIH-SUS; SINAN; SINITOX; SIOPS; SISAB; SISPRENATAL; Site-TB; Sivep-gripe; SNGPC; VIGITEL; SAMMED; and VIVA BEM - APP
*Regional (province, state, more than one city)*	AGHU; APURASUS; BNAFAR; CIHA; CMD; COAGULOPATIAS; Conecte SUS - APP; DATATOX; e-SUS AB; Farmácia Cidadã; FARMÁCIA POPULAR; HORUS; Micronutrientes; NOTIVISA/VIGIMED; PERIweb; PNAUM; PNS; S-CODES; SAMMED; SI-PNI; SIAFI; SIASI; SIA-SUS; SICLOM; SIGAF; SIH-SUS; SINAN; SINITOX; SIOPS; SISAB; SISPRENATAL; Site-TB; Sivep-gripe; SNGPC; VIGITEL; and VIVA BEM - APP
*Municipality (one city)*	None
*Organization multi-sited*	None
***Sector of data source***	
*Public health system*	AGHU; APURASUS; BNAFAR; CMD; COAGULOPATIAS; e-SUS AB; Farmácia Cidadã; FARMÁCIA POPULAR; HORUS; Micronutrientes; SAMMED; SIAFI; SIASG/SISME; SIASI; SIA-SUS; SICLOM; SIGAF; SIH-SUS; SISAB; SISPRENATAL; Site-TB; VIGITEL; and VIVA BEM - APP
*Private sector*	SNGPC
*Both*	BPS; CIHA; Conecte SUS - APP; DATATOX; NOTIVISA/VIGIMED; PERIweb; PNAUM; PNS; S-CODES; SAMMED; SI-PNI; SINAN; SINITOX; SIOPS; Sivep-gripe; and VIGITEL
***Type of data source***	
*Wholesaler*	None
*Pharmacy records*	BNAFAR; CIHA; COAGULOPATIAS; Conecte SUS - APP; Farmácia Cidadã; FARMÁCIA POPULAR; HORUS; Micronutrientes; SI-PNI; SIAFI; SIASG/SISME; SIASI; SIA-SUS; SICLOM; SIGAF; SISAB; SNGPC; and VIVA BEM - APP
*Patient records*	AGHU; CIHA; COAGULOPATIAS; Conecte SUS - APP; e-SUS AB; SAMMED; SIASI; SIA-SUS; SIH-SUS; SISAB; SISPRENATAL; Site-TB; and VIVA BEM - APP
***Setting of data source***	
*Ambulatorial*	BNAFAR; COAGULOPATIAS; e-SUS AB; Farmácia Cidadã; FARMÁCIA POPULAR; HORUS; Micronutrientes; SI-PNI; SIA-SUS; SIGAF; SISAB; and SNGPC
*Hospital*	SIH-SUS
*Both*	AGHU; APURASUS; CIHA; CMD; Conecte SUS - APP; NOTIVISA/VIGIMED; PERIweb; S-CODES; SAMMED; SIASI; SICLOM; SINAN; SINITOX; SIOPS; SISPRENATAL; Sivep-gripe; and VIVA BEM - APP
***Type of data***	
*Aggregate level* ^b^	APURASUS; BPS; CIHA; COAGULOPATIAS; DATATOX; Micronutrientes; PNS; S-CODES; SAMMED; SI-PNI; SIAFI; SIASG/SISME; SIASI; SIA-SUS; SICLOM; SIH-SUS; SINAN; SINITOX; SIOPS; SISAB; SISPRENATAL; SNGPC; and VIGITEL
*Individual level*	AGHU; BNAFAR; CIHA; Conecte SUS - APP; e-SUS AB; Farmácia Cidadã; HORUS; Micronutrientes; NOTIVISA/VIGIMED; PERIweb; PNAUM; PNS; SIA-SUS; SIGAF; SIH-SUS; SINAN; Site-TB; Sivep-gripe; SNGPC; and VIVA BEM - APP
***Years coverage***	
Since	1979–2020

a Data sources can be classified in more than one category within the same domain.

b Data sources that provide aggregate level data and also figure as individual level can be available for research after requesting data for custodians and/or ethical approval.

URLs for the “publicly and conveniently accessible online” data sources are shown in Table 2, as well as the file formats. The URLs for all data sources selected are provided in the supplemental material (Supplementary Table S5). Access through the FTP directory is provided for limited data sources and is also provided in the supplemental material (Supplementary Table S6).

**TABLE 2:** Additional characteristics: path and file format available among data sources freely available online.

Data source	Custodian	Path	File Format
*BPS*	Ministry of Health	https://antigo.saude.gov.br/gestao-do-sus/economia-da-saude/banco-de-precos-em-saude/bases-anuais-compiladas	CSV (ZIP)
*CIHA*	Ministry of Health	http://ciha.datasus.gov.br/CIHA/index.php	DBC
CMD*	Ministry of Health	http://datasus.saude.gov.br/transferencia-de-arquivos2/#; https://conjuntominimo.saude.gov.br/#/cmd; http://www2.datasus.gov.br/DATASUS/index.php?area=0901&item=1&acao=37	CSV
*Micronutrientes*	Ministry of Health	https://sisaps.saude.gov.br/micronutrientes/	CSV
*PNAUM*	Ministry of Health	http://www.ufrgs.br/pnaum	TXT (ZIP)
*PNS*	Ministry of Health	http://www2.datasus.gov.br/DATASUS/index.php?area = 0208&id = 28247790; https://www.ibge.gov.br/estatisticas/sociais/justica-e-seguranca.html	HTML
		https://dados.gov.br/dataset/xn-pesquisa-nacional-de-saude	DBC
		—	JSON
		—	XML
		—	ODS
*SI-PNI*	Ministry of Health	http://pni.datasus.gov.br/; https://datasus.saude.gov.br/acesso-a-informacao/imunizacoes-desde-1994/	XLS
*SIA-SUS*	Ministry of Health	http://www2.datasus.gov.br/DATASUS/index.php?area = 0901	DBC
*SIH-SUS*	Ministry of Health	http://www2.datasus.gov.br/DATASUS/index.php?area = 0901	DBC
*SINAN*	Ministry of Health	https://portalsinan.saude.gov.br/dados-epidemiologicos-sinan	CSV
		http://datasus.saude.gov.br/transferencia-de-arquivos2/#	DBC
*SINITOX*	Oswaldo Cruz Foundation (Fiocruz)	https://sinitox.icict.fiocruz.br/dados-regionais	PDF
*SIOPS*	Ministry of Health	http://siops.datasus.gov.br/relUN.php?acao = 7	HTML
*SISAB*	Ministry of Health	https://sisab.saude.gov.br/index.xhtml	Excel
			CSV
			ODS
*SISPRENATAL*	Ministry of Health	http://datasus1.saude.gov.br/sistemas-e-aplicativos/epidemiologicos/sisprenatal	DBC
		http://datasus.saude.gov.br/transferencia-de-arquivos2/#	
*Sivep-gripe*	Ministry of Health	http://plataforma.saude.gov.br/coronavirus/dados-abertos/	CSV
*SNGPC*	Brazilian Health Regulatory Agency (Anvisa)	https://dados.gov.br/dataset?q = sngpc&sort = score + desc%2C + metadata_modified + desc	CSV
*VIGITEL*	Ministry of Health	http://datasus.saude.gov.br/vigitel-vigilancia-de-fatores-de-risco-e-protecao-para-doencas-cronicas-por-inquerito-telefonico/	XLS
		http://svs.aids.gov.br/download/Vigitel/	

In Brazil, six different ways of assigning codes to medicines were found. Drug coding is not uniform; each data source has its own coding system, depending on the purpose of the data. The drug coding systems employed in Brazil are shown in Table 3, with examples of data sources that use each system. This information was not available for all data sources, an indication of the need for further work on characterization.

**TABLE 3:** Drug coding system in Brazil.

Drug coding system	Description	Data sources
EAN-13	This is the International Article Number (also known as European Article Number or EAN). It is a standard describing a barcode symbology and numbering system used in global trade to identify a specific retail product type, in a specific packaging configuration, from a specific manufacturer. In Brazil, it presents the National Code of the Products	FARMÁCIA POPULAR; BPS
CATMAT	This is the Material Registry of the Ministry of Economy (in Portuguese, Cadastro de Materiais do Ministério da Economia). This code allows the cataloging of materials destined to the activities and means of Public Administration. The categories referring to health products and medicines are under the responsibility of the Cataloging Unit of the Ministry of Health (UC/MS). The objective is to establish and maintain a unique and standardized language for the identification, coding and description of materials to be acquired by the Federal Government, through ComprasNet.	BNAFAR; HORUS; BPS
DCB	The Common Brazilian Denomination (in Portuguese, Denominação Comum Brasileira) is the medication name according to the National List of Essential Medicines (in Portuguese, RENAME, Relação Nacional de Medicamentos Essenciais). It is the generic name (non-proprietary or non-commercial) of the drug or pharmacologically active principle, based on the official chemical name and pharmacological classification, and approved by the Thematic Technical Committee of the Brazilian Pharmacopoeia Commission (CTT-DCB), in the form of Board Resolution Anvisa Collegiate Body (RDC)	BPS; SI-PNI; SISAB; CMD
SIGTAP	This the code adopted by the System of Procedures, Medicines and OPM Management of the Unified Health System. It is known as the SUS Table (in Portuguese, *Tabela* SUS)	SIA-SUS; SIH-SUS; SISAB
Register number	This is the register number that informs the complete number by which the product is registered with *Anvisa*, including the digits related to the presentation (13 digits)	BPS
Specific codes, drug name, or active principle	Name of the medication recorded as the name of active principle or coded according to the study protocol	PNAUM; PNS; SGNPC; SINAN

The literature review was part of the initial process for mapping Brazilian databases. We found publications related to 23 of the 38 data sources, including reports, manuals, and other documents available online. Scientific articles had been published in national and in international journals. Examples of studies that used some of the selected data sources are presented in Table 4.

**TABLE 4:** Examples of DUR published using Brazilian data sources.

Title	Data source
*Acesso e uso de medicamentos para hipertensão arterial no Brasil* Mengue et al. (2016b)	PNAUM
*Uso de medicamentos e outros produtos com finalidade terapêutica entre crianças no Brasil* Pizzol et al. (2016)	
*Prevalência da automedicação no Brasil e fatores associados* Arrais et al. (2016)	
*Utilização de anti-hipertensivos e antidiabéticos no Brasil: análise das diferenças socioeconômicas. Pesquisa Nacional de Saúde 2013* Monteiro et al. (2019)	PNS
*Análise clínica e epidemiológica das internações hospitalares de idosos decorrentes de intoxicações e efeitos adversos de medicamentos, Brasil, de 2004 a 2008* Paula et al. (2012)	SINITOX; SIH-SUS
*Sistema nacional de informações tóxico-farmacológicas: o desafio da padronização dos dados* Santana et al. (2011)	
Bortoletto and Bochner (1999)	
*Eventos adversos notificados ao Sistema Nacional de Notificações para a Vigilância Sanitária (NOTIVISA): Brasil, estudo descritivo no período 2006 a 2011* Oliveira et al. (2013)	NOTIVISA/VIGIMED
*Reações adversas a medicamentos no sistema de farmacovigilância do Brasil, 2008 a 2013: estudo descritivo* Mota et al. (2019)	
*Record linkage of pharmacovigilance and registration databases: a study of biological medicines in Brazil* Soares and Silva (2013)	
*Perfil da utilização de antimicrobianos em um hospital privado* Rodrigues and Bertoldi (2010)	AGHU
*Ações judiciais: estratégia da indústria farmacêutica para introdução de novos medicamentos* Chieffi and Barata (2010)	S-CODES
*Evaluation of a web-based registry of inherited bleeding disorders: a descriptive study of the Brazilian experience with HEMOVIDAweb Coagulopatias* Rezende et al. (2017)	Coagulopatias
*Quality Evaluation of Poison Control Information Systems: A Case Study of the DATATOX System* Alves et al. (2016)	Datatox
*Aspectos relacionados à utilização de antirretrovirais em pacientes de alta complexidade no estado do Rio de Janeiro, Brasil* Madruga et al. (2018)	SICLOM
*Utilização do e-SUS AB e fatores associados ao registro de procedimentos e consultas da atenção básica nos municípios brasileiros* Thum et al. (2019)	SISAB
*Farmácia Cidadã: Integralidade, Humanização e Racionalidade Na Atenção Ao Paciente* Machado-dos-Santos (2014)	Farmácia Cidadã
*Programa “Farmácia Popular do Brasil”: caracterização e evolução entre 2004–2012* Silva and Caetano (2015)	FARMÁCIA POPULAR
*Gastos com pagamentos no Programa Aqui Tem Farmácia Popular: evolução entre 2006–2014* Silva and Caetano (2018)	
*Towards preventive pharmacovigilance through medicine misuse identification: an example with recombinant human growth hormone for aesthetic purposes* Rodrigues-Neto et al. (2018)	PERIweb
*Vigitel Brasil: vigilância de fatores de risco e proteção para doenças crônicas por inquérito telefônico: estimativas sobre frequência e distribuição sociodemográfica do uso e fontes de obtenção dos medicamentos para tratamento da hipertensão e diabetes nas capitais dos 26 estados brasileiros e no Distrito Federal, 2011 a 2013* Ministério da Saúde (2017)	VIGITEL
*Evaluation study of the National Immunization Program Information System* Silva et al. (2018)	SI-PNI
*Evidências advindas do consumo de medicamentos moduladores do apetite no Brasil: um estudo farmacoeconométrico* Mota and Silva (2012)	SNGPC
*Consumo do benzodiazepínico clonazepam (Rivotril®) no estado do Rio de Janeiro, Brasil, 2009–2013: estudo ecológico* Zorzanelli et al. (2019)	
*Uso de registros de assistência farmacêutica do Sistema de Informações Ambulatorial para avaliação longitudinal de utilização e adesão a medicamentos* Soares and Silva (2013)	SIH/SIA-SUS
*Costs in the Treatment of Schizophrenia in Adults Receiving Atypical Antipsychotics: An 11-Year Cohort in Brazil* Barbosa et al. (2018)	
*Ten-year kidney transplant survival of cyclosporine- or tacrolimus-treated patients in Brazil* Gomes et al. (2016)	
*Demographics, deaths and severity indicators in hospitalizations due to drug poisoning among children under age five in Brazil* Maior et al. (2020)	
*Public financing of human insulins in Brazil: 2009–2017* dos Santos Dias et al. (2020)	SIASG
*Immunosuppressants in Brazil: underlying drivers of spending trends, 2010–2015* Alves et al. (2018)	

## Discussion

This study provides an overview of data sources that are used or have the potential to be used for DUR in Brazil. A total of 38 sources were identified, almost half of which are publicly available and provide national information. Nineteen sources collect individual-level data, but few provide it for download. Those classified as “other sources” were generally related to Ministry of Health administrative processes, as medicines purchases and prices. Further characterization to determine the types of research questions they might address is needed. In Brazil, six different ways of assigning codes to medicines are employed, none of which is recognized internationally. Brazilian data sources have the potential to answer research questions related to medication use, adherence to treatments, purchases, and safety. However, currently mapped sources comprise a mix of databases, of unknown quality, centralized by the national government, but decentralized in terms of research and their usability and purposes for decision-making and post-market surveillance.

Some of the data sources presented here had been used by researchers. Ali et al. have described the linkable databases currently available for evaluating health technology assessment in Brazil (Ali et al., 2019). For example, the CIDACS initiative uses SINAN, SIH-SUS, SINASC (Live Births Information System), and SIM (Mortality Information System) to assess outcomes of major social programs (Barreto et al., 2019). SIM (not included in our inventory because it presents only data related to ICD-10 codes for drug poisoning mortality (Mota et al., 2012)) and SINASC are important sources of data for evaluating health outcomes and indicators. The quality of data in both systems has improved over time (Szwarcwald et al., 2019; França et al., 2020); however, health outcomes of medication exposure (not related to poisoning) remain unexplored for most classes of medicines.

Junior et al. linked SIH, SIA, SIM, SINASC and SINAN (Guerra Junior et al., 2018) and created a National Database of Health for longitudinal studies. Freire et al. linked SIM and SIH, including information from APACs (Authorization of High Complexity Procedures of the Outpatient), provided by the SIA-SUS system, and were able to describe the trajectory of patients in the health care network, and cancer-related hospital admissions (Freire et al., 2015). In fact, the APAC reports are among the most important sources of information on medication dispensing in Brazil. However, the information pertains only to drugs dispensed free of charge; that is, only medications supplied by SUS under the APACs are recorded and available through DataSus systems. Moreover, the generation and consolidation of APACs to make the data available for DUR are complex. Few research groups have the expertise required to link the different data sources and prepare the data for longitudinal analysis (Soares and Silva, 2013).

Exposure to medications among the Brazilian population is complicated by the structure of health care delivery, where a private system co-exists with a public system, and no overall control is in place for dispensing most medicines. Consequently, only studies using data from APACs for biological agents, chemotherapy, and other high-cost medicines have the potential to correctly ascertain exposure (Prestes, 2017; Junior et al., 2018).

Other automated health care databases, some of which were identified by Ali et al. (Ali et al., 2019), could be valuable for DUR, but not without an extensive evaluation of the quality of the data they contain. Notable examples are *Horus* *, Farmacia Popular* and BNAFAR. Interfaces among the systems that generate these databases are known, but nothing is known about their quality, coverage, and completeness. These data sources, specifically the BNAFAR and the *Horus,* were not available for research (Ministério da Saúde, 2018). Infrastructure issues are familiar limitations, and at least partially explain why data on drug dispensing are so difficult to obtain in our country (Herrett et al., 2015; Hallas et al., 2017). Pharmacoepidemiology research perspectives in Brazil suffer constraints not due to lack of data, but to lack of linked data and cross-validated secondary data (de Castro, 1999; Junior et al., 2018; da Saúde, 2018).

The *Sistema Nacional de Gerenciamento de Produtos Controlados* (SNGPC) (Agência Nacional de Vigilância Sanitária, 2019)**,** which monitors dispensing of narcotic and psychotropic medications, and since 2013, antibiotics, is an important data source for controlling the purchase and dispensing of medicines. An “open data” initiative launched by ANVISA has yielded data for DUR. The expectation is that data provided by ANVISA might allow assessing, for example, policy impact of medicines regulation. However, a complete characterization of these data sources for understanding the quality of provided data, and what research questions would be answered using the open data are still lacking.

SIVEP-Gripe and SI-PNI, among other automated health care databases (Table 1), record information on medication use, but the quality, temporality and feasibility for linkage of these data have not been adequately explored for DUR. SIVEP-Gripe is available and provides individual-level data, but the incompleteness of certain variables and lack of temporality in recording medication use, render the information useless for examining, for instance, the effectiveness of medication use. SIVEP-Gripe is an epidemiologic surveillance system that was designed for other purposes, but with properly recorded information, it could help answer important research questions and support other voluntary reporting systems in evaluating adverse drug effects (Melo et al., 2021). As well, non-prescription drugs recorded in surveillance systems such as SINAN, and SIVEP-Gripe are often taken during the onset of a disease—an upturn in sales may serve as an early indicator of an outbreak or epidemic (Das et al., 2005; Edge et al., 2006).

The Electronic Medical Record (EMR) of the Management Application for University Hospitals–AGHU currently covers 30 hospitals across the country (Ministério da Educação, 2019). It is the standard management system for all federal university hospitals provided by the *Empresa Brasileira de Serviços Hospitalares* (Ebserh) network and is a potential data source for DUR. University hospitals treat both in- and outpatients. The creation of a large cohort of patients receiving different levels of care would allow for follow-up of short- and long-term effects of medication on several outcomes. e-SUS AB might be used for the same purpose. However, no single DUR study was found to have used the Ebserh data.

We classified four data sources as adverse event report systems: NOTIVISA/VIGIMED, SINAN, SINITOX, and DATATOX. Recently, ANVISA published implementation of the VigiFlow (named Vigimed in Brazil) (Vogler et al., 2020) as a substitute for the NOTIVISA in an effort to enhance the usability of the national system. But no information is available about how different pharmacovigilance systems across the country could be integrated. In 2021, part of Vigimed aggregated data was available on the Anvisa website by drug, adverse reaction (MedDRA SOC/Preferred Term), severity, age group, gender, state of the case report, for example. Clinical trial reports are also recorded in the same database (Notivisa EC) but are not available given the need for data confidentiality.

Spontaneous reporting systems constitute a major resource for detecting adverse drug effects and have made important contributions to pharmacoepidemiology (Strom and Carson, 1990). Systems for active surveillance and projects for detecting signals and monitoring recently approved medications (Racoosin et al., 2012) have been established in other countries. Recent studies involving disproportionality analysis for safety signal screening in children (Vieira et al., 2020) and breast cancer patients (Barcelos et al., 2019) using Notivisa were conducted, demonstrating the potential of this data source. However, Brazil lags behind in terms of research initiatives and decision-making using automated administrative data.

The only national-level drug utilization study that has been conducted in Brazil was based on primary data (Mengue et al., 2016a). The National Survey on Access, Use and Promotion of Rational Use of Medicines (PNAUM) was a cross-sectional, population-based study focusing on urban households. Fieldwork was carried out between September 2013 and February 2014. In total, 41,433 interviews were carried out. The survey examined medication use for chronic health conditions. However, the PNAUM has not been repeated, and the cross-sectional data do not allow evaluation of outcomes. Also, this was the only study to collect population-level data about over-the-counter medication use. Currently, no information about over-the-counter is available in any of the automated databases (Arrais et al., 2016). Other important surveys (cross-sectional) were included in our inventory—PNAD and Vigitel—although their purpose is to assess other characteristics of the Brazilian population and do not provide medication details.

Brazil has no formal policy on setting priorities and using administrative data to evaluate the effectiveness and safety of medications. However, many systems contain information for managing logistics and drug expenditures. APURASUS, SIGAF and SIASG are used by different levels of government to control costs and transmit information from local systems to the national level to plan acquisition and distribution. For example, SIASG made it possible to explore expenditures, pricing and judicial demands for a variety of drugs and drug classes, and it has been important for decision-making about the incorporation of drugs in the national list and the sustainability of provision programs (Luo et al., 2014; Chaves et al., 2017; Chama Borges Luz et al., 2017; Magarinos-Torres et al., 2017; dos Santos Teodoro et al., 2017; Alves et al., 2018; Caetano et al., 2020; dos Santos Dias et al., 2020, 2009–2017; Matos et al., 2020). However, the safety profile of medicines and outcomes in the population cannot be examined with these data.

Despite efforts made by the Ministry of Health to harmonize the recording of information, health institutions’ data collection processes differ considerably. Because of the structure of the healthcare system, patients typically seek care from a variety of providers at several institutions with nonlinked electronic health record systems. Combining data from these systems is a challenge. One of the most important issues to emerge from this study is the lack of unique key identifiers for individuals. These factors, in addition to technological infrastructure and skilled human resource constraints, limit the usefulness of routinely collected data in generating evidence to support clinical and policy decisions and in answering epidemiological questions (Ali et al., 2019).

Another important finding is the heterogeneity of drug-coding systems in Brazil. Federal Law No. 9,787/99 requires that, within the scope of the SUS, purchases of medicines, under any type of acquisition, as well as medical and dental prescriptions for medicines, adopt the DCB (Brazilian Non-proprietary name) or, in their absence, the International Non-proprietary Name (INN). However, this does not apply to administrative databases. For each data source, it is necessary to know the types of codes that are employed, how they are constructed, and why they are used, but no clear definitions are provided.

The limitations of this inventory of Brazilian databases that contain medication-related information are mainly related to the design of the study and the difficulty of assembling a group of experts with an in-depth knowledge of each data source. We may have missed data sources and relevant studies. The literature search was conducted using the names of the data sources, but if a name was unknown, studies could not be found, and the data source was not included. Moreover, this is only an inventory; full characterization of each database has yet to be done.

The main value of this study is to provide an overview with a focus on data sources for DUR. The methodology used by the LatAm project may be highlighted as one of the main strengths of our study, an original multi-phase approach allowing to map national data sources for DUR. The next step is to fully characterize each database using pre-established checklists (Hall et al., 2012), and thereby provide information that will help researchers determine which sources may be may be useful for specific types of studies; what research questions can feasibly be addressed; how the data can be accessed; and what quality may be expected from the data.

Based on this comprehensive and structured inventory, we provided an overview of the several types of data sources for DUR in Brazil. Our findings demonstrated that a uniform system for drug classification, data quality evaluation, and the extent of population covered by year are lacking in the mapped data sources. National administrative health databases are provided mainly through the DataSus and contain information about the population covered by the SUS. Further work is required to assess the reliability of Brazilian data for DUR.

## Data Availability

The original contributions presented in the study are included in the article/Supplementary Materials, further inquiries can be directed to the corresponding author.

## Glossary

(alphabetic order, Portuguese acronyms and names) AGHU: Aplicativo de Gestão para Hospitais Universitários
APURASUS: Sistema de Apuração e Gestão de Custos do SUS
BNAFAR: Base Nacional de Dados de Ações e Serviços da Assistência Farmacêutica no SUS
BPS: Banco de Preços em Saúde
CIHA: Sistema de Comunicação de Informação Hospitalar e Ambulatorial
CMD: Conjunto Mínimo de Dados
Datatox: Sistema Brasileiro de Dados de Intoxicações
e-SUS AB: e-SUS Atenção Básica
HÓRUS: Sistema Nacional de Gestão da Assistência Farmacêutica
NOTIVISA/VIGIMED: Sistema de Notificação em Vigilância Sanitária
PERIweb: Sistema de Notificação Espontânea de Suspeita de Reação Adversa a Medicamento ou Desvio da Qualidade de Medicamento do Estado de São Paulo
PNAU: Pesquisa Nacional sobre Acesso, Utilização e Promoção do Uso Racional de Medicamentos no Brasil
PNS: Pesquisa Nacional de Saúde
S-CODES: Sistema de Coordenação de Demandas Estratégicas–SP
SAMMED: Sistema de Acompanhamento do Mercado de Medicamentos
SIAFI: WebService Sistema Integrado de Administração Financeira
SIASG/SISME: Sistema Integrado de Administração de Serviços Gerais/Sistema de Minuta de Empenho
SIASI: Sistema de Informação da Atenção da Saúde Indígena
SIA-SUS: Sistema de Informações Ambulatoriais do SUS
SICLOM: Sistema Gerencial de Controle Logístico de Medicamentos
SIGAF: Sistema Integrado de Gerenciamento da Assistência Farmacêutica
SIH-SUS: Sistema de Informação Hospitalar
SINAN: Sistema de Informação de Agravos de Notificação
SINITOX: Sistema Nacional de Informações Tóxico-Farmacológicas
SIOPS: Sistema de Informações sobre Orçamentos Públicos em saúde
SI-PNI: Sistema de Informações do Programa Nacional de Imunizações
SISAB: Sistema de Informação em Saúde para a Atenção Básica
SISPRENATAL: Sistema de acompanhamento do programa de humanização no pré natal e nascimento
SITE-TB: Sistema de Informação de Tratamentos Especiais de Tuberculose
SIVEP-Gripe: Sistema de informação de vigilância epidemiológica da gripe
SNGPC: Sistema Nacional de Gerenciamento de Produtos Controlados
VIGITEL: Vigilância de fatores de risco e proteção para doenças crônicas por inquérito telefônico
